# Just say know: Drug education and its publics in 1980s Britain

**DOI:** 10.1016/j.drugpo.2020.103029

**Published:** 2021-02

**Authors:** Alex Mold

**Affiliations:** Centre for History in Public Health, London School of Hygiene & Tropical Medicine, 15-17 Tavistock Place, London, WC1H 9SH, United Kingdom

**Keywords:** Heroin, Drug education, Health education, History of drug use

## Abstract

Until the 1980s, anti-drug education campaigns in the UK were rare. This article examines the reasons behind a policy shift that led to the introduction of mass media drug education in the mid 1980s. It focuses on two campaigns. ‘Heroin Screws You Up’ ran in England, and ‘Choose Life Not Drugs’ ran in Scotland. The campaigns were different in tone, with ‘Heroin Screws You Up’ making use of fear and ‘shock horror’ tactics, whereas ‘Choose Life Not Drugs’ attempted to deliver a more positive health message. ‘Heroin Screws You Up’ was criticised by many experts for its stigmatising approach. ‘Choose Life Not Drugs’ was more favourably received, but both campaigns ran into difficulties with the wider public. The messages of these campaigns were appropriated and deliberately subverted by some audiences. This historical policy analysis points towards a complex and nuanced relationship between drug education campaigns and their audiences, which raises wider questions about health education and its ‘publics’.

In April 1986, the cast of teen TV soap, *Grange Hill*, released a song titled ‘Just say no’. The single reached number five in the charts and remained in the Top 100 for five weeks. It delivered a clear anti-drug use message, telling listeners to ‘just say no’ to drugs. The song built on a recent storyline in which one of the characters, Zammo McGuire, became addicted to heroin. ‘Just say no’ also echoed American First Lady Nancy Reagan's campaign of the same name, and an anti-drug use TV advertisement made by the Scottish Health Education Group in 1985. The premise of such ‘just say no’ messages was that telling children and young people about the dangers of drugs would dissuade them from substance use. Like other health education programmes during the 1980s, the intention behind drug education was to increase knowledge about drugs, provoke behaviour change and encourage individuals to make healthier choices. The use of such campaigns to prevent drug use was, however, controversial. Health educators and experts on drug use had long advised against mass drug education efforts, for fear that these would have the opposite effect. Many authorities believed that drug education campaigns would provide information about drugs to young people that they were previously unaware of, increasing the likelihood that they would take drugs. Concerns about the so-called ‘boomerang effect’ meant that there were very few anti-drug campaigns in Britain until the 1980s (Manning, 2013).

This article explores the reasons behind the change in drug education policy during the 1980s and examines two campaigns to tease out some wider issues around drug education and its publics. An increase in heroin use and growing media attention meant that the government wanted to be seen to take action on drugs, leading to the introduction of mass-media campaigns, even though this went against expert advice. The ‘Heroin Screws You Up’ campaign, which ran in England from 1985 until 1986 and the ‘Choose Life Not Drugs’ campaign which ran in Scotland over the same period, were very different in tone. The ‘Heroin Screws You Up’ campaign made use of fear and ‘shock horror’ tactics; in contrast the ‘Choose Life Not Drugs’ campaign situated its anti-drug message within a broader positive health agenda. ‘Heroin Screws You Up’ was widely criticised at the time, whereas ‘Choose Life Not Drugs’ was received more warmly, but both campaigns were open to alternative readings and re-appropriation by their audiences. Anti-drug use messages were interpreted in a variety of ways by different ‘publics’. These readings were shaped by the beliefs and behaviours of groups and individuals, but also influenced by social structures, economic circumstances and political framings.

To address such issues, this article focuses on some of the visual materials produced as part of these campaigns and how they were received. Such an approach has provided a fruitful line of analysis for historians of other public health issues (Cooter & Stein, 2010; Hand, 2017; Medcalf & Nunes, 2018). It is especially pertinent here for two reasons. Firstly, there is a lack of archival material relating to the development of the anti-heroin campaigns and the strategies behind them. This is not that unusual; campaign materials may survive but documents relating to their production often does not. Secondly, the visual materials facilitate an exploration of the relationship between intention and reception, between public health actors and their publics. This is important, as it broadens the focus away from the issue of whether or not such campaigns ‘work’. Since the late 1970s, health educators and their paymasters questioned the effectiveness of their efforts in changing individual behaviour and generating healthier outcomes. A more recent meta-analysis of health education campaigns suggests that whilst there is evidence of some successes, especially around smoking, most only produce a moderate effect (Wakefield, Loken & Hornik, 2010). That does not mean however, that such campaigns are unworthy of study. A ‘failed’ campaign can tell us as much, if not more, than a successful one. By looking at two such campaigns this article draws out a set of wider issues about how these materials were read and interpreted by different audiences, the relationship between experts and policy makers, and the political and symbolic value of health education efforts even when these do not achieve their putative goals.

## Drug use and health education, 1960s–1980s

Educating people about dangers to health had long been part of public health practice, but this took on a new level of importance in the second half of the twentieth century. As chronic disease linked to lifestyle became the leading cause of morbidity and mortality, emphasis was placed on informing people about health risks and persuading them to change their behaviour. Mass media health education campaigns were increasingly common from the 1960s onwards. In 1964, the Cohen committee on health education recommended that greater use be made of the mass media not only to provide health information, but also to change behaviour (Ministry of Health, 1964). In the wake of the Cohen committee the Health Education Council (HEC) was set up in 1968 in England and Wales, and the Scottish Health Education Group (SHEG) in Scotland. Numerous campaigns on various issues were launched by these groups, on topics including obesity, alcohol and the dangers posed by smoking (Berridge, 2007; Berridge & Loughlin, 2005; Hand, 2020; Mold, 2017). By the early 1980s, however, doubts about the ability of such campaigns to change behaviour and reduce harmful outcomes were beginning to arise. An editorial published in the *British Medical Journal* in 1982 argued that the HEC had achieved little (Anon, 1982). Some health educators stressed the importance of social context and rejected a sole focus on individual behaviour change (Rodmell & Watt, 1986). In the UK and at the global level, a wider focus on ‘health promotion’, rather than simply ‘health education’ was put forward (Duncan, 2013; Kickbusch, 2003). This encompassed the environmental, social and economic determinants of health and advocated for a set of policies that went beyond health education in order to improve public health.

In addition to questions about the overall effectiveness of health education campaigns, there were specific reasons why anti-drug use campaigns had not been attempted in Britain. Manning argues that the lack of drug education campaigns prior to the 1980s was rooted in the British approach to drugs which was primarily medical, unlike the US, which framed drug use in moral terms (Manning, 2013). But there were more prosaic elements at work too. Although cannabis and LSD use increased during the 1960s and 1970s, the use of ‘hard’ drugs like heroin and cocaine was relatively rare. The number of what were called ‘known heroin addicts’ (those who were notified to the Home Office) did not exceed 2000 until the end of the 1970s (Advisory Council on the Misuse of Drugs, 1983). There was also considerable professional opposition to drug education campaigns, from both health educators and drug use experts alike. A leaflet on young people and drugs produced by the HEC and the Institute for the Study of Drug Dependence (ISDD) in 1975 was a rare exception, and even this was aimed primarily at parents. The leaflet stated that ‘We know it is not any use talking at young people, nor trying to scare them by depicting horrific consequences’ (Institute for the Study of Drug Dependence & Health Education Council, 1975). Indeed, there was some evidence of substance awareness campaigns in the US leading to an increase in drug use, the so-called ‘boomerang effect’ (Haskins, 1979; Swisher, Crawford, Goldstein & Yura, 1971). In the UK, in 1979, the Central Office of Information (COI) echoed such fears warning that an ‘ill-advised approach by feeding interest in drugs in the wrong way, may actually encourage experimentation’ (Central Office of Information, 1979, p. 17).

However, when heroin use started to increase, the value of drug education was re-evaluated. In 1977, there were 2016 ‘known heroin addicts’, by 1982 there were 4371 (Advisory Council on the Misuse of Drugs, 1983). In 1984, the government's expert body on drugs, the Advisory Council on the Misuse of Drugs (ACMD), examined the issue of health education campaigns. They asserted that ‘education aimed specifically at preventing drug misuse is not yet common because of the fear that it will promote experimentation’ (Advisory Council on the Misuse of Drugs, 1984, p. 20). The ACMD warned against ‘shock and horror’ tactics, noting that campaigns ‘based on such measures on their own are likely to be ineffective or, at worst, positively harmful’ (Advisory Council on the Misuse of Drugs, 1984, pp. 35–36). The Council also raised questions about the whole premise of drug education, noting that ‘we do not accept that educational programmes based only on the provision of accurate factual information about drugs and their potential dangers are sufficient to prevent drug misuse. It is now generally accepted that knowledge of itself does not usually change attitudes, far less alter behaviour’ (Advisory Council on the Misuse of Drugs, 1984, p. 36). Instead, the ACMD argued that drug education was better placed within a broader programme of health education messaging aimed at promoting a healthy lifestyle – taking exercise, not smoking, eating a good diet, and so on.

By the early 1980s, the rising number of people using heroin, and a growing moral and media panic around heroin use, made a health education campaign politically expedient, even if it was not well-supported amongst experts. In 1983 there were 5079 ‘known heroin addicts’, by 1987 there were 10,389, with many thousands more going undetected. At the same time, social and economic conditions facilitated the development of problematic drug use and heightened the sense of fear that surrounded this. Mass unemployment, particularly amongst young people, and high levels of deprivation were linked to rising levels of drug use, especially in urban areas (MacGregor, 1989). Popular and media presentations of young people tended to portray them as either vulnerable or violent, perceptions that were strongly influenced by notions of race, ethnicity and class (Connell, 2019; Peplow, 2019). Increasing drug use amongst young people was one of a number of social problems that demanded political and practical attention.

## Heroin screws you up

Given this context, it is unsurprising that the impetus for the ‘Heroin Screws You Up’ campaign came from government ministers. The Department of Health and Social Security (DHSS) initially asked the HEC if they would run an anti-heroin campaign. The HEC were reluctant, as a specific education campaign to tackle drug use went against the advice of health educators and drug experts. The HEC also wanted time to gather information and to develop materials, as well as additional resources, but the government demanded swift action (House of Commons Social Services Select Committee, 1985). Instead, the DHSS turned to the COI, the government's official communications body, to run the campaign. The COI commissioned the advertising agency Yellowhammer to develop the campaign, where it was designed by Sammy Harai, who went on to produce the famous AIDS education campaign featuring a tombstone and the voice of John Hurt a few years later.

In the absence of a full archival record, the strategy behind ‘Heroin Screws You Up’ can be gleaned from what was said about it at the time and from the materials themselves. A report on the campaign stated that it was intended to ‘prevent further increases in the prevalence of drug misuse, and ultimately to reduce its incidence, especially where heroin is concerned’ (Research Bureau International, 1986). According to the Conservative party health minister Ray Whitney, the aim of the campaign was to influence and inform young people deemed to be ‘most at risk’ (Whitney, 1986). These were defined as: young people who had been involved in the ‘misuse of other drugs but not heroin’; or who were ‘living in areas where heroin use prevailed’; where there were ‘adverse social and economic circumstances’; or they had ‘friends using heroin’ (Andrew Irving Associates, 1986). The identification of the target audience was thus related to a particular construction of the population ‘at risk’ that was rooted in wider social and economic issues. But, the mitigation of such risks was a task for the individual, not for government. As with other health education campaigns at the time, this was intended to make those thought to be at risk or engaging in risky behaviours responsible for dealing with these (Lupton, 1995).

**Fig. 1:**
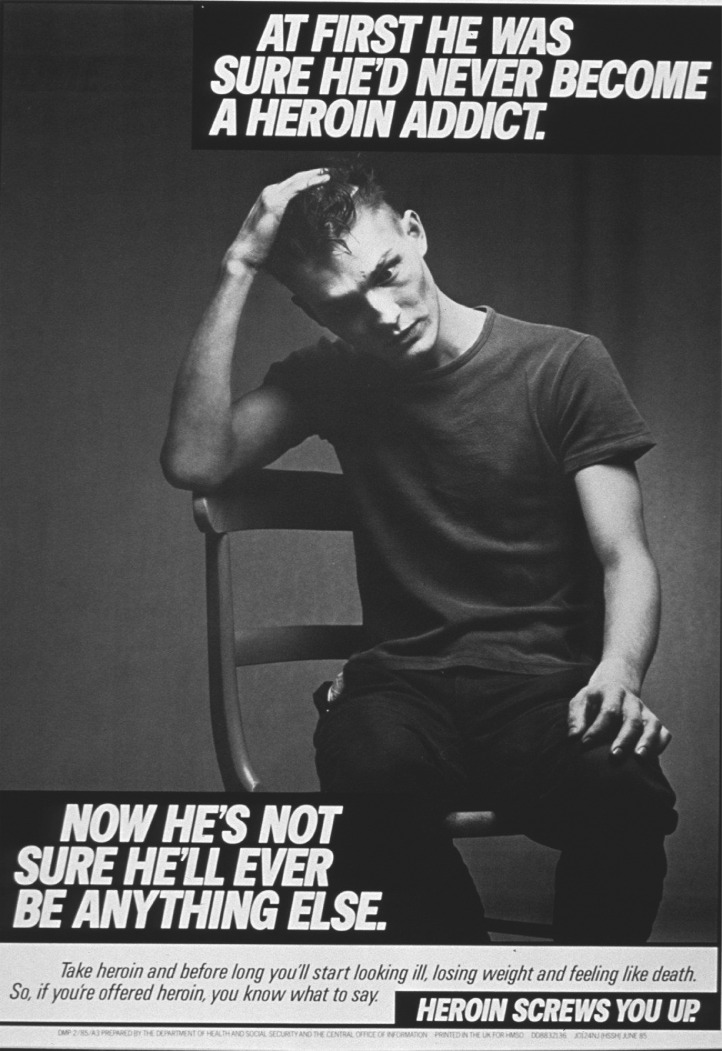
‘At first he was sure he'd never become a heroin addict’. Department of Health and Social Security and the Central Office of Information 1985.

The ‘Heroin Screws You Up’ campaign consisted of two short TV films, a series of press and magazine advertisements, a poster and a leaflet for parents. It ran from April 1985 to March 1986, and it cost £2 million. The magazine and newspaper advertisements were mostly black and white. This meant that they were cheaper to reproduce, but the monotone colouring also exaggerated the dark message of the advertisements and the dangerousness of heroin. The advertisements featured a mixture of male and female protagonists, all were young, and all were white. Each one of the different images had a similar look, but a slightly different emphasis, presumably designed to reach different audiences. Many of the images focused on the physical and mental consequences of heroin use. In ‘Your mind isn't the only thing heroin damages’ the viewer is confronted with a black and white image of a young man seated in a hunched over position. He appears to be pale, sweaty, dirty and has spots on his skin. Superimposed on top of the image are seven text labels. These catalogue the physical effects of injecting drug use and addiction to heroin, including skin infections, blood diseases, liver complaints and ‘mental problems.’ The text underneath the image offers a narrative of progressive decline and loss of control, from use to sickness and addiction. Similar tropes were at work in other images from the campaign. In ‘At first he was sure he'd never become a heroin addict. Now he's not sure he'll ever be anything else’ we find another pale and gaunt young man, this time seated on a chair with his hand on his head in a seemingly distraught manner. The text underneath states that ‘Take heroin and before long you'll start looking ill, losing weight and feeling like death. So if you're offered heroin, you know what to say. Heroin screws you up.’ [Fig. 1] This message emphasises the apparent inevitability of addiction, even for those who are sure they can ‘handle it’.

The theme of loss of control and progressive decline intersected with other tropes in ‘How much is heroin likely to cost you’. The advertisement features a succession of images of the same young woman, moving from left to right across the page. In the first image she appears healthy and confident, with her hand on her hip and looking directly into the camera. In the second image, she is standing but looking downwards. In the third image, the woman is seated on a chair, her hair appears to be greasy and she has dark circles around her eyes. In the final image she is seated on the floor, hunched over with a hand pulling at her hair. Superimposed on top of each image is a short text label indicating what heroin will cost the user: ‘It'll cost you your friends’, ‘Your looks’, ‘Your possessions’ and ‘Your health’. The text underneath the image echoes the narrative of progressive decline, but also emphasises the price of heroin use in relation to the loss of possessions, as well as the danger to health. ‘How much will heroin cost you’, and ‘Skin care by heroin’ were clearly targeted at young women, denoted by the images of women and the fact that unlike the ones targeted at young men, these advertisements placed greater emphasis on the risk heroin use posed to physical appearance, an issue presumably thought to be of more appeal to women. The gendered dimensions of this campaign also sat alongside an emphasis on the effect heroin might have on a teenagers’ possessions and their ability to acquire new ones. Such tactics were representative of the place of the teenager (itself a relatively new construction) in a burgeoning consumer society.

The dark tone of the campaign and its consumerist framing influenced the ways in which it was received. The campaign was evaluated for the DHSS and the COI by two commercial market research companies. One agency carried out a quantitative study. They surveyed 700 young people aged between 13 and 20, both before and after the campaign. The agency found that 95% of respondents had heard of the campaign. Moreover, the survey suggested that there was significant change after the campaign in relation to awareness about the health risks associated with heroin use. Respondents reported being less likely to take heroin if offered it by a friend after having seen the campaign. This was the case with even with those deemed most ‘at risk’. The campaign appeared to have reached its target group, but the reception of its message was more problematic. The survey noted an increased belief that death was an inevitable consequence of heroin usage (Research Bureau International, 1986). This was erroneous, but it was perhaps not surprising that respondents believed this, given the tone of the campaign.

A qualitative study, based on focus groups and in-depth interviews, dug more deeply into the attitudes, beliefs and behaviours of the target audience. This also found high levels of recall of the campaign and its message, especially with those thought to be most ‘at risk’ from using heroin. The evaluators asserted that the campaign had fostered and reinforced negative attitudes and beliefs about heroin misuse across the sample. They also pointed to an apparent increased confidence amongst what the report called heroin ‘rejectors’ by providing them with more information about the downsides of using the drug. However, the report also sounded notes of caution. Some of those most ‘at risk’ (people who knew someone who already used drugs) were less likely to say that heroin was more dangerous than cannabis after the campaign than before it. The authors also felt that ‘the current commercials and press advertisements show signs of wear out: if young people become bored with the executions, they may begin to become more resistant to and react against the message.’ The effects of the campaign were likely to be short-term, and ‘long-term effects might be less favourable unless future activity is handled with care and is both sensitive and responsive to the changing attitudes amongst young people’ (Andrew Irving Associates, 1986).

Notwithstanding these warnings, the campaign's paymasters appeared happy with the results. Health minister Whitney pointed to the high levels of recall amongst respondents and the suggestion that attitudes towards heroin were more cautious amongst some young people after the campaign as a sign of its success. He asserted that although unrealistic claims for the effect of the campaign should not be made, the results were ‘encouraging’ (Whitney, 1986). Yet the campaign was criticised by those working in the drugs field, the wider media and even an internal DHSS review. Questions were raised about the methods used in the evaluation of the campaign, with a number of critics pointing to the small sample size. This mattered especially in relation to the assertion that a change in attitudes had occurred after the campaign (DHSS, n.d.). But it was the campaign itself that provoked the most ire. Some critics saw the use of fear tactics to scare young people into not using drugs as something which could increase the stigmatisation around drugs and drug users, but also that such images would not be credible to those more familiar with drugs (Rhodes, 1990). Others felt that the campaigns were too broadly targeted, unlikely to achieve behaviour change, that attitudes towards drugs were influenced by a range of other media, and embedded within broader cultural and social structures and values (Hansen, 1985; Power, 1989; Woodcock, 1986). More worryingly, scare tactics might have encouraged young people to use drugs as an act of rebellion (Falk-Whynes, 1991).

It is impossible to know if the ‘Heroin Screws You Up’ campaign led to an increase in drug use, but there is evidence to suggest that some young people deliberately appropriated the campaign and its imagery. There were numerous accounts of teenagers taking the poster or the magazine and newspaper advertisements and putting them up on their bedroom walls, not in support of the message, but in an ironic commentary on it (Ashton, 2002, p. 137). In 1989, Barry Sheerman (Labour MP for Huddersfield East) told the House of Commons that ‘The rather effete young man in the heroin posters became a pin-up for some young girls.’ Conservative Health Minister David Mellor responded that this was an allegation that had never been proven (House of Commons, 1989). Irrespective of the extent to which this happened the imagery the campaign featured was clearly open to wider cultural appropriation or re-appropriation. This is an example of the ‘polysemic’ nature of ‘texts’: that these can be read in multiple ways, some of which may be in direct opposition to that which the creators intended (Miller, Kitzinger, Williams, & Beharrell, 1998, pp. 210–211). In the case of ‘Heroin Screws You Up’, this went beyond the immediate context of the campaign. In the mid-1990s, androgynous models began to appear on catwalks and in magazines with emaciated features, pale skin and dark circles underneath the eyes. This look was branded ‘heroin chic’ (Arnold, 1999; Harold, 1999; Hickman, 2002). Visually, there were many common tropes with those portrayed in the ‘Heroin Screws You Up’ campaign. Whilst there were a whole host of other elements behind the creation of ‘heroin chic’, the similarity between this look and the health education campaign highlights the fact that imagery created for one purpose in one context is not owned by any one group or fit for one purpose.

## Choose life not drugs

**Fig. 2:**
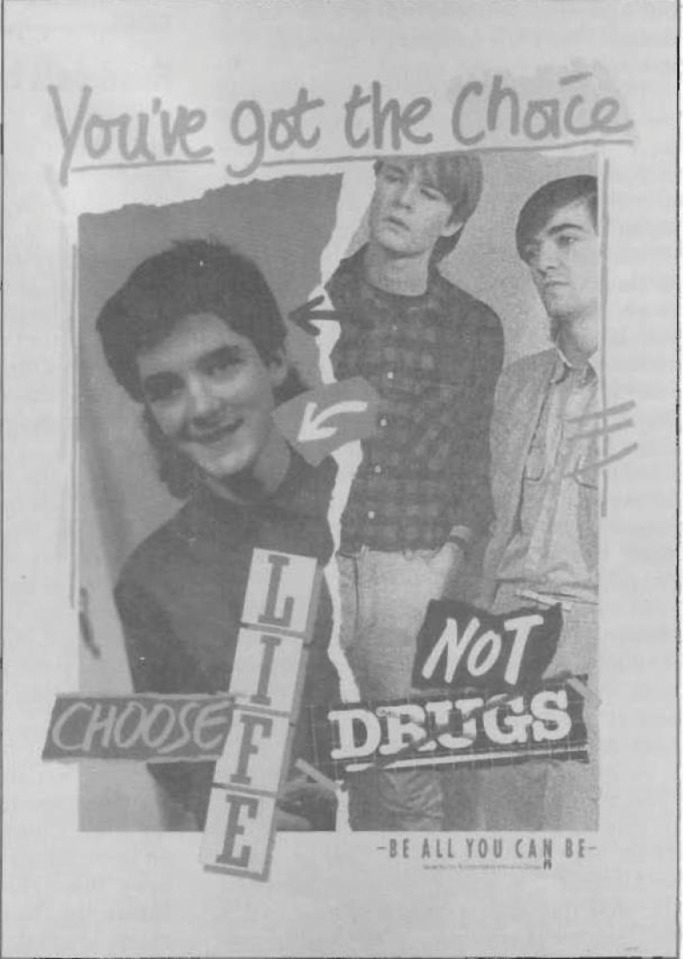
Choose Life Not Drugs Scottish Health Education Group, 1985.

The multiple meanings of drug education messages was not confined to the ‘Heroin Screws You Up Campaign’. Although the Scottish Health Education Group's (SHEG) 1984–85 campaign, ‘Choose Life Not Drugs’, was very different in tone, its public reception was also problematic. SHEG were instructed to mount an anti-drug use campaign by the Scottish Office, and although they were reluctant to do so, they agreed as long as it could be located within their existing work which promoted positive health (Davies, 1988; Whitehead, 1989). The campaign, which cost £350,000, was launched in March 1985 (Jagger, 1986). Like ‘Heroin Screws You Up’, it consisted of TV advertisements and magazine and newspaper inserts. The tone and style of the material, however, was very different. The four TV advertisements were built around positive messages, including ‘Be All You Can Be’, ‘Choose Life Not Drugs’ and ‘Just Say No’. They were made in the style of a ‘pop video’ and emphasised building young people's self-esteem (Whitehead, 1989). According to Jagger, the ‘Choose Life Not Drugs’ film was based on a decision making model, where individuals were confronted with situations where they might be encouraged to use drugs, such as peer group pressure, but they chose to respond ‘positively’ (Jagger, 1986). Raymond suggested that the film promoted the notion that young people could make their own decisions despite socio-economic constraints (Raymond, 1989). The magazine advertisement echoed the message about the ability to make positive choices. The advertisement was titled ‘You've got the choice. Choose life not drugs. Be all you can be’. It displayed a photograph of a smiling youth on one side of the picture, and two less-happy looking young men on the other side. Arrows moving between the two images suggested the correct direction of travel: making the ‘right’ choices resulted in happiness and well-being [Fig. 2].

The SHEG campaign material was an explicit rejection of the ‘shock horror’ tactics deployed in ‘Heroin Screws You Up’. The difference in tone between the campaigns was not a reflection of the different health systems in Scotland and England, but rather a result of the fact that health educators ran the ‘Choose Life’ campaign, whereas the ‘Heroin Screws You Up’ campaign was designed by a professional advertising agency at the behest of the COI. Given the fact that the ‘Choose Life’ campaign was designed by health educators it is no surprise that it was received more warmly by health educators than the ‘Heroin Screws You Up’ campaign. ‘Choose Life’ was even praised at a World Conference on Health Education in 1985 (Coyle, 2008, p. 212). Yet, surveys of respondents pointed to some issues with the campaign's impact and interpretation. Awareness of the campaign was high. A survey conducted by the Advertising Research Unit at the University of Strathclyde found that 67% of 13–16-year olds, and 77% of 17–20-year olds, had heard of the campaign. Nearly all of the respondents felt that it promoted positive alternatives to drugs, but a third of those surveyed believed that the campaign treated the issue too light-heartedly. More significantly, a quarter of the sample thought that the campaign was ‘out of touch with reality’, and a quarter of working class and unemployed teenagers saw it as ‘just a pretence’ (Davies, 1988, p. 24). An especially problematic element was the framing of drug use as a ‘choice’. The ‘choose life’ message was open to a range of different interpretations and readings, some of which ran counter to that which was originally intended.

Indeed, the ‘choose life’ slogan already had another set of connotations. ‘Choose life’ was emblazoned across numerous t-shirts throughout the 1980s. These were launched in 1983 by the fashion designer Katherine Hamnett. She said that ‘Choose life’ was inspired by a Buddhist expression, and was one of number of political messages put on t-shirts by Hamnett, but the ‘Choose life’ t-shirts were particularly popular – especially after they were worn by pop group Wham. A more direct inversion of the ‘choose life’ message in relation to drugs appeared a few years after the campaign. In the 1993 book, and 1996 film, *Trainspotting*, the central character, Mark Renton, a heroin user, delivers a soliloquy on the hollowness of ‘choosing life’ and how he chose not to choose life, but chose heroin instead. Irvine Welsh, the author of the book, deliberately inverted the ‘choose life not drugs’ message of the 1985 campaign to say precisely the opposite. In the 2017 film sequel, *Trainspotting 2*, Renton even says as much when a new character asks him what ‘choose life’ means: He says, ‘"Choose life" was a well-meaning slogan from a 1980s antidrug campaign. And we used to add things to it’ (Hodge, 2017).

Renton, of course, was not alone in choosing not to choose life. Indeed, large sections of the public appeared to be reluctant to make the healthy choices that they were being exhorted, encouraged or cajoled towards. A key piece of research was conducted in South Wales in the mid-1980s. In an evaluation of a health promotion campaign intended to inform the public about the risks of developing heart disease, researchers found that beliefs about heart disease and risk were made up of a mixture of official messages interwoven with ideas derived from the mass media and the experiences of friends and family. Indeed, these were crucial to how people understood risk and thus how they responded to health education campaigns. The team noted that: ‘[*a*]n aged and healthy friend, acquaintance or relative – an “Uncle Norman” – who has smoked heavily for years, eats a diet rich in cream cakes and chips and/or drinks ‘like a fish’ is a real or imagined part of many social networks … A single Uncle Norman, it seems, may be worth an entire volume of medical statistics and several million pounds of official advertising’ (Davison, 1989). Uncle Norman, and a degree of fatalism about the inevitability of sickness and death, allowed people to continue to behave in ways that they knew had negative health consequences. Some members of the public could resist health promotion messages when these did not chime with their lived experiences or ran counter to other kinds of desires. If healthy living could be framed as a choice, then so was unhealthy living (Lupton, 1995).

Other research at the time and since called into question the language of choice in such settings. Were unhealthy behaviours really choices? If so, what factors shaped them? Looking at why the public continued to make unhealthy choices exposed a range of reasons, both individual and structural. In a now classic study of young mothers who smoked, sociologist Hilary Graham found that for the women she spoke to, smoking was a way of coping with poverty and the demands of motherhood (Graham, 1987). Smoking, as was increasingly obvious by the 1980s, was strongly correlated with socioeconomic status, with the poorest in society the most likely to smoke. Other kinds of negative health behaviours, from obesity to drug taking, often followed a similar pattern (Daniel et al., 2009; McLaren, 2007). The reasons for this are complex, but at the very least this problematises the notion of choice. The ability to make choices is even further undermined in situations where there may be an element of dependence involved (Brook, 2010). The persistence of behaviour-related public health problems cannot simply be ascribed to individual choice (Cornell & Milner, 1996; Mayes, 2015; Petersen, Davis, Fraser & Lindsay, 2010). Such ‘choices’ were shaped by factors beyond the control of the individual.

## Conclusion

Considering the role of social, political and environmental structures in shaping individual behaviours highlights the importance of placing drug education campaigns and the response to these in context. This historical policy analysis has pointed to the contested origins of the ‘Heroin Screws You Up’ and ‘Choose Life Not Drugs’ campaigns. Both were introduced primarily as a reaction to rising heroin use and the need to be seen to take action. Health educators and experts on drug use were largely against these campaigns. Many of their fears were realised in the reception of the campaigns. This was especially the case with ‘Heroin Screws You Up’, where the ‘shock and horror’ approach added to the stigmatisation of drugs and drug users. Although the ‘Choose Life Not Drugs’ campaign was more positive in tone, and somewhat more in line with the views of health educators at the time, the ambivalent reaction of some audiences pointed to a wider disconnect between the message and those supposed to be receiving it. Indeed, the re-appropriation of imagery and text from both campaigns and its re-purposing in a variety of ways denotes a dynamic relationship between public health messages and their ‘publics’ (Mold, Clark, Millward & Payling, 2019). Such publics were not merely passive recipients. They could do much more than either accept or reject the message: they could actively re-interpret it so that it came to acquire a new set of meanings, sometimes in direct opposition to the original. This operated not just in relation to the campaign materials, but also to the broader underpinning concept of choice. ‘Choice’ was inevitably constrained by circumstance, but this did not mean that individuals lacked agency. It was possible to make ‘unhealthy’ choices, to choose the ‘wrong’ things.

‘Heroin Screws You Up’ and ‘Choose Life’ may not have ‘worked’ in the sense that they failed to achieve lasting behaviour change or prevented many young people from using drugs, but they ‘worked’ as a way to demonstrate to a wider public that the government was taking action to deal with drug use. This narrative could be turned on its head by some viewers, and the campaigns also ‘worked’ as a way to voice teenage rebellion. This was then further appropriated by fashion designers, novelists and film makers. Not all of this was foreseen by health educators at the time, but they did caution against such efforts for fear that these would backfire. Politicians ignored expert advice as they had other objectives in mind. This was not necessarily ‘wrong’, as policymakers must balance a range of issues and interests, but it does highlight a distinction between expert advice and policy objectives. In the wake of the Covid-19 pandemic, the relationship between politicians, policy makers, experts and health education messaging has come in for renewed scrutiny. This historical analysis thus serves as a useful reminder not only that mass media health education campaigns are fraught with difficulty, but also that these can throw light on a broader set of issues about values, the role of experts in policy making and practice, and the complex relations between government and the governed.

## Declaration of Interests

I have no competing interests.
